# Discriminative Validity of Field-Based Propulsion and Sprint Tests in Elite Wheelchair Court Athletes with Different Functional Profiles

**DOI:** 10.3390/sports14060220

**Published:** 2026-05-28

**Authors:** Jordi Sanchez-Grau, Roger Font, Víctor Toro-Román, Gerard Carmona, Adrián García-Fresneda

**Affiliations:** 1 Faculty of Health Sciences, San Jorge University, Autov. A-23 Zaragoza-Huesca, KM 299, 50830 Villanueva de Gállego, Spain; jsanchezgr@tecnocampus.cat; 2 Research Group in Technology Applied to High Performance and Health (TAARS), Tecnocampus, Department of Health Sciences, Pompeu Fabra University, 08302 Mataró, Spain; rfont@tecnocampus.cat (R.F.); vtoro@tecnocampus.cat (V.T.-R.); fresneda@tecnocampus.cat (A.G.-F.)

**Keywords:** paralympic sport, propulsion, functional capacity, wheelchair court sports, functional classification, wheelchair mobility

## Abstract

**Purpose**: Field-based tests are widely used to assess propulsion and sprint performance in wheelchair athletes; however, their ability to discriminate between functional performance profiles associated with different impairment characteristics remains insufficiently explored. This study evaluated the discriminative capacity of propulsion, sprint, and manoeuvrability tests in elite wheelchair court athletes. **Methods**: Nineteen male elite athletes (ten wheelchair basketball, nine wheelchair rugby) performed the initial maximum push-rim propulsion (IMPRP), a 12 m linear sprint (3, 5, and 12 m splits), and a wheelchair manoeuvrability test (3L3R). Test reliability was assessed using intraclass correlation coefficients (ICC). **Results**: Test reliability was high across all assessments (ICC ≥ 0.82). The higher functional performance profile group demonstrated substantially greater IMPRP mechanical outputs, including mean velocity (ES = 2.69), maximum velocity (ES = 3.29), mean power (ES = 1.75), and maximum power (ES = 2.09) (all *p* < 0.001). Sprint performance also showed large between-group differences at 5 m (ES = 1.53) and 12 m (ES = 1.68) (*p* < 0.001), whereas manoeuvrability differences were moderate (ES = 0.62; *p* = 0.043). **Conclusions**: IMPRP and short-distance sprint tests appeared sensitive to differences between ecologically distinct wheelchair court sport athletes characterised by different real-world functional performance profiles. These field-based assessments may be useful for performance monitoring and may complement ecologically distinct athlete groups in wheelchair court sports.

## 1. Introduction

Wheelchair court sports, such as wheelchair basketball (WB) and wheelchair rugby (WR), are highly demanding Paralympic disciplines that require athletes to repeatedly combine propulsion, acceleration, and agility with precise technical and tactical execution [1,2,3,4,5]. These sports involve repeated high-intensity wheelchair propulsion interspersed with rapid directional changes and contact situations, placing considerable demands on upper-limb strength, trunk stability, and wheelchair handling skills.

Although WB and WR share similar playing environments and propulsion mechanics, athletes competing in these sports typically present different functional impairment profiles, which influence their capacity to generate propulsion force, accelerate, and manoeuver the wheelchair efficiently.

In Paralympic sport, classification aims to ensure fair competition by grouping athletes according to functional capacity—that is, the extent to which an athlete’s impairment affects sport-specific performance—rather than by medical diagnosis alone [6]. In WB, the International Wheelchair Basketball Federation assigns classification scores ranging from 1.0 (most limited functional capacity) to 4.5 (greatest functional capacity) [7], with teams on court restricted to a combined total of 14.0 points [8]. WB athletes typically present lower-limb impairments (e.g., spinal cord injuries, amputations, or musculoskeletal dysfunctions) that may limit mobility but often allow relatively preserved trunk control and upper-limb function.

By contrast, WR follows a stricter classification system defined by the World Wheelchair Rugby Federation (WWR), from 0.5 (minimal functional capacity) to 3.5 (maximal functional capacity) [9], with teams of four players limited to a combined total of 8.0 points [10]. WR athletes commonly present impairments affecting all four limbs—frequently associated with cervical spinal cord injuries, multiple amputations, or neuromuscular conditions resulting in tetraplegia—which can substantially reduce trunk stability, hand function, and propulsion capacity.

As a consequence, elite WB and WR athletes often represent ecologically distinct athlete groups characterised by different real-world functional performance profiles within wheelchair court sports, particularly with regard to trunk control, upper-limb strength, and wheelchair propulsion efficiency [11]. These differences in athlete characteristics provide an opportunity to examine whether commonly used field-based performance tests are sensitive to performance differences between ecologically valid wheelchair court sport groups.

Field-based tests such as the initial maximum push-rim propulsion (IMPRP) [12] and short-distance linear sprints [10,13,14] have been widely used to evaluate propulsion mechanics and acceleration capacity in wheelchair athletes [15]. These tests are practical, time-efficient, and easily implemented in applied sport environments. Previous research has demonstrated good reliability for these assessments and their relevance for monitoring training adaptations and physical performance in wheelchair court athletes.

However, the discriminative validity of these tests across athletes presenting different functional impairment profiles remains insufficiently explored. Establishing whether propulsion and sprint tests can distinguish between athletes with markedly different functional capabilities is important for several reasons. First, it provides insight into the sensitivity of these tests for performance monitoring. Second, it may help identify performance indicators associated with wheelchair propulsion capacity in ecologically valid wheelchair court sport contexts. Finally, such information may contribute to the development of more evidence-informed approaches to athlete performance monitoring in wheelchair court sports [16].

Therefore, the aim of this study was to evaluate the discriminative capacity of field-based propulsion, sprint, and manoeuvrability tests in elite wheelchair court athletes presenting different functional performance profiles. Elite WB and WR players were examined as representatives of two distinct functional profiles commonly observed in wheelchair court sports. It was hypothesised that propulsion and sprint tests would demonstrate strong discriminative capacity between the sampled athlete groups, whereas manoeuvrability performance would show smaller between-group differences.

## 2. Materials and Methods

### 2.1. Design

A descriptive cross-sectional design was used to evaluate the discriminative capacity of field-based wheelchair performance tests across elite wheelchair court athletes presenting different functional performance profiles [17]. Athletes completed the initial maximum push-rim propulsion (IMPRP), a 12 m linear wheeling sprint (3, 5, and 12 m splits), and a wheelchair manoeuvrability test (3L3R). The IMPRP assessed maximal single-push propulsion output from a stationary position, whereas the 12 m sprint and 3L3R assessed linear acceleration and directional agility, respectively.

To assess reproducibility, the intraclass correlation coefficient (ICC), coefficient of variation (CV), and standard error of measurement (SEM) were calculated for each test. To quantify the magnitude of between-profile differences and the practical discriminative value of each test, between-group comparisons were performed, and standardised mean differences (effect sizes, ES) were calculated.

Although participants were drawn from two sports (WB and WR), the analytical purpose was not to compare sports per se, but to examine whether these commonly used field tests can discriminate between athletes typically presenting different real functional and sport-specific performance profiles within wheelchair court sport.

### 2.2. Subjects

A total of nineteen elite wheelchair athletes voluntarily participated in this study, including ten WB players (age = 27.8 ± 9.8 years; total mass = 83.0 ± 17.3 kg)—seven from the Spanish national team and three competing in the first Spanish national league—and nine WR players from the Spanish national team (age = 35.3 ± 8.1 years; total mass = 83.5 ± 14.0 kg). All participants were registered members of the Spanish Sports Federation for Physical Disabilities (FEDDF) and were officially classified according to the guidelines of the International Wheelchair Basketball Federation (IWBF) and World Wheelchair Rugby (WWR) classification committees (Table 1).

Because WB and WR use different classification scales and athletes typically present different impairment distributions, classification scores were not directly compared across sports. Instead, groups were interpreted as representing two distinct functional performance profiles commonly observed in wheelchair court sports. Consequently, between-group comparisons should be interpreted as test discrimination between ecologically distinct athlete groups, not as equivalence or differences between classification categories.

To minimise potential confounding by training exposure, testing was scheduled during the same competitive period (transitional Christmas period, 2023/2024) and within the same week for both groups. Both teams reported a comparable weekly training structure, consisting of 2–3 wheelchair court sessions (technical/tactical) and 2 strength and conditioning sessions per week. Nevertheless, because WB and WR differ in sport-specific demands, wheelchair configuration, and propulsion strategies, these factors may also have contributed to the observed between-group differences. Therefore, the findings should be interpreted within the context of ecologically valid wheelchair court sport performance profiles rather than as isolated indicators of impairment-related functional capacity.

Before data collection, no participant reported injury in the preceding month, and all were considered fit to perform maximal testing. The institutional research ethics committee of the Catalan Sports Council approved the study (015_CEICGC_2023). Written informed consent was obtained from all participants in accordance with the Declaration of Helsinki (2013).

### 2.3. Procedures

Testing comprised IMPRP, linear wheeling sprint, and wheelchair manoeuvrability (3L3R). All tests were conducted indoors on the same surface (parquet). Participants were familiarised with procedures beforehand and refrained from strenuous exercise for ≥48 h prior to testing. Each team was tested collectively on the same day, and both teams were tested within the same week.

Before each test, all participants performed a standardised 10 min warm-up described elsewhere [18], consisting of continuous wheeling, joint mobility exercises, progressive submaximal accelerations (for the wheelchair manoeuvrability and sprint tests), and five short accelerations (prior to the IMPRP).

Athletes performed all tests using their own sport-specific wheelchairs (WB or WR). This approach maximised ecological validity but also implies that outcomes reflect the combined contribution of the athlete’s functional profile and sport-specific wheelchair configuration. Therefore, the results are interpreted with respect to the discriminative capacity of the field tests under real-world conditions rather than as isolated physiological comparisons between sports.

#### 2.3.1. Initial Maximum Push-Rim Propulsion

The IMPRP has been standardised and is increasingly applied in WB and WR athletes of both sexes, as previously described [12]. Briefly, the protocol consists of two repetitions separated by a 30 s passive rest interval. From a stationary position, athletes perform simultaneous bilateral arm action to generate a single maximal push on the wheelchair rims, after which they return to the starting position. Performance was measured using a linear encoder (Chronojump Boscosystem, Barcelona, Spain) (accuracy: 61 mm; sampling rate: 1000 Hz) attached via a strap to the horizontal axis between the push wheels, with data recorded through Chronojump free software (version 2.6.0) configured to compute displacement and time in a linear plane [12,19,20,21]. The total system mass (athlete plus wheelchair) was entered into the software to calculate mechanical outputs, including mean velocity (V), maximum velocity (Vmax), mean power (P), relative mean power (Rel. P), maximum power (Pmax), relative maximum power (Rel. Pmax), mean force (F), relative mean force (Rel. F), maximum force (Fmax), and relative maximum force (Rel. Fmax). Each repetition was terminated when force production decreased to zero [12]. Both repetitions were considered for the reliability analysis, while the best trial was retained for subsequent analyses.

#### 2.3.2. Linear Wheeling Sprint

Linear sprint performance was assessed using two 12 m wheelchair sprints performed at maximal speed, separated by a 2 min passive rest interval [19]. At the start of each sprint, participants positioned themselves 0.5 m behind the starting line and initiated the trial when ready. They then propelled their wheelchairs forward and were verbally encouraged to exert maximal effort. Performance was measured using a Race Analyzer (Chronojump, BoscoSystem, Barcelona, Spain) configured at 10 pulses per sample, corresponding to a spatial accuracy of 3.032 cm. Each sample recorded the elapsed time between pulses with a temporal precision of 4 microseconds [22]. The device was operated by a researcher seated 2 m behind the participants and was attached to the wheelchair at the horizontal frame bar. Each velocity–time curve for the 3, 5, and 12 m sprint tests was fitted post hoc by a mono-exponential function using least squares regression:

(1) $$v_{h\left(t\right)}=\;v_{h,max}\cdot \left(1\;-\;e^{\left\{-\tau /t\right\}}\right)$$

After integration of *v_h(t)_* (Equation (1)), the horizontal displacement of the wheelchair system (proxy for whole-body centre of mass) can be expressed as:

(2) $$x\left(t\right)=\;v_{max}\cdot \left(\;t\;+\;\tau \cdot e^{-t/\tau }\right)-\;v_{\left\{max\right\}}\cdot \;\tau$$

Sprint times at 3, 5, and 12 m were extracted from the modelled displacement–time curves. Both repetitions were included in the reliability analysis, and the fastest trial was retained for subsequent analyses.

#### 2.3.3. Wheelchair Manoeuvrability Test (3L3R)

**Figure 1:**
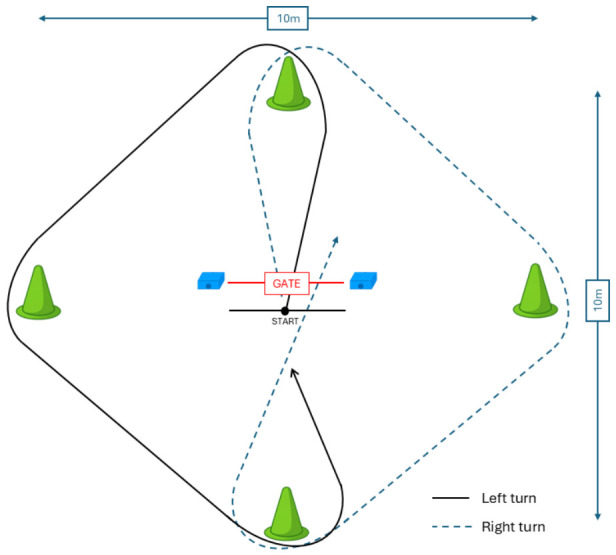
Three Left Turns and Three Right Turns (3L3R) test. The solid black line indicates the first left turn, while the dotted blue line indicates the second right turn, facilitating understanding of the spatial sequence of the route.

The Three Left Turns and Three Right Turns (3L3R) test was used to assess wheelchair manoeuvrability (directional agility). Each participant completed two maximal-effort trials, separated by a 2 min passive recovery period, and was verbally encouraged to perform at their highest intensity. Performance time (s) was recorded using a pair of infrared photocells (ChronoJump, BoscoSystem, Barcelona, Spain) positioned 0.40 m above the ground at the start/finish gate. Participants began 0.5 m behind the starting line and initiated movement at a self-selected moment following the start signal to avoid reaction-time bias. Upon crossing the gate for the first time, athletes executed a left turn, which triggered the first split time. They then performed a right turn, crossing the gate again to register the second split, and continued alternating turns until completing a total of three left and three right turns (Figure 1). For subsequent analyses, the fastest trial was retained, determined by the shortest total completion time, calculated as the sum of all left and right split times.

### 2.4. Statistical Analyses

The reproducibility of the performance tests was evaluated using the intraclass correlation coefficient (ICC), coefficient of variation (CV = [SD/mean] × 100), and the standard error of measurement (SEM) to assess both relative and absolute reliability [23]. For applied interpretation, the ICC was considered the primary indicator of test reproducibility, whereas the CV and SEM were used as complementary metrics to describe relative variability and absolute measurement error. The distribution of each variable was examined, and the normality of continuous variables was assessed using the Shapiro–Wilk test. Differences between WB and WR players in IMPRP mechanical outputs, linear wheeling sprint performance, and 3L3R performance were evaluated using independent samples t-tests for normally distributed data or the Mann–Whitney U test for variables violating the assumption of normality. Standardised mean differences (Cohen’s effect size [ES]) were calculated, with thresholds interpreted as follows: <0.20, trivial; 0.20–0.59, small; 0.60–1.19, moderate; 1.20–1.99, large; and ≥2.0, very large [24].

All analyses were performed using the Statistical Package for the Social Sciences (SPSS™, version 25.0 for Mac, Chicago, IL, USA), and the significance level was set at *p* < 0.05.

All inferential comparisons are reported as differences between groups representing distinct functional performance profiles, acknowledging that sport-specific wheelchair configuration is embedded within these profiles.

## 3. Results

Table 2 and Table 3 summarise the mean values and reliability indices (ICC, CV, SEM) for the IMPRP, sprint tests (3, 5, 12 m), and the 3L3R manoeuvrability test in WB and WR players.

Overall, both groups demonstrated good to excellent relative reliability across all tests. ICC values were generally ≥0.82, with sprint and manoeuvrability measures showing particularly high reproducibility. Absolute reliability, reflected by the CV and SEM, was low for most variables. No significant differences were observed between Trial 1 and Trial 2 for any measure.

Reliability analyses demonstrated excellent reproducibility across all field-based tests (ICC = 0.82–0.99; CV generally <11%), supporting the suitability of these assessments for evaluating propulsion and mobility performance in elite wheelchair court athletes.

**Figure 2:**
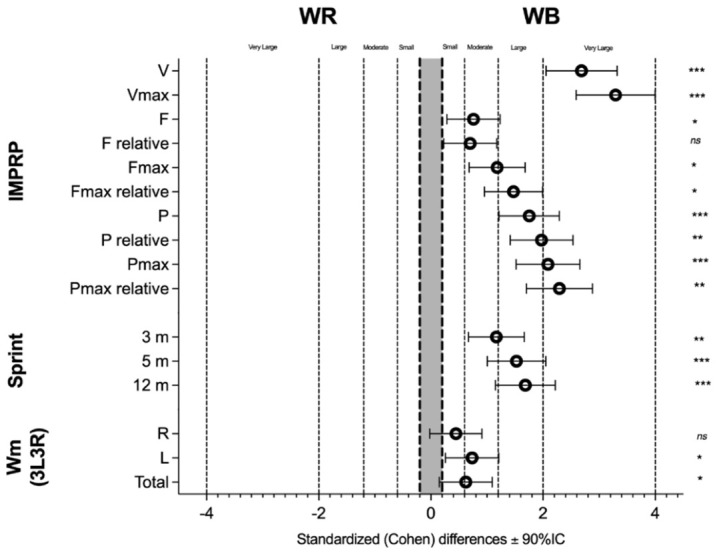
Standardised (Cohen’s *d*) differences ± 90% confidence intervals for initial maximum push-rim propulsion (IMPRP), sprint time intervals, and wheelchair manoeuvrability test (Wm) (3L3R) comparing two sampled wheelchair court athlete groups (wheelchair basketball [WB] and wheelchair rugby [WR]). F, mean force; Fmax, maximum force; F relative (N·kg^−1^); Fmax relative (N·kg^−1^); P, mean power; Pmax, maximum power; P relative (W·kg^−1^); Pmax relative (W·kg^−1^); V, mean velocity; Vmax, maximum velocity; R, right; L, left. Statistical significance: ns = not significant; * *p* < 0.05; ** *p* < 0.01; *** *p* < 0.001.

Significant differences in physical performance outcomes were observed between the two athlete groups representing distinct functional performance profiles across the three assessed tests (Figure 2). This figure represents the effect size, providing information about the observable differences between groups, where values closer to 0 indicate smaller between-group differences, whereas values farther from 0 indicate greater differences between groups.

In the IMPRP test, athletes representing higher functional performance profiles demonstrated substantially greater mechanical propulsion outputs. Specifically, the higher functional profile group (WB) achieved significantly higher mean velocity (mean ± SD = 1.181 ± 0.2 m·s^−1^ vs. 0.732 ± 0.126 m·s^−1^; *p* < 0.001; ES = 2.69) and maximum velocity (2.382 ± 0.238 m·s^−1^ vs. 1.398 ± 0.349 m·s^−1^; *p* < 0.001; ES = 3.29). Similarly, the higher functional profile group produced significantly greater mean power (234.325 ± 63.031 W vs. 130.944 ± 54.664 W; *p* < 0.001; ES = 1.75), relative mean power (3.02 ± 1.293 W·kg^−1^ vs. 1.293 ± 1.051 W·kg^−1^; *p* = 0.002; ES = 1.97), maximum power (722.988 ± 263.888 W vs. 255.933 ± 174.635 W; *p* < 0.001; ES = 2.09), and relative maximum power (8.633 ± 2.451 W·kg^−1^ vs. 2.64 ± 2.77 W·kg^−1^; *p* < 0.001; ES = 2.29). The very large effect sizes observed across most IMPRP variables indicate that this test shows strong sensitivity for distinguishing propulsion capacity between athlete groups presenting different functional performance characteristics.

**Figure 3:**
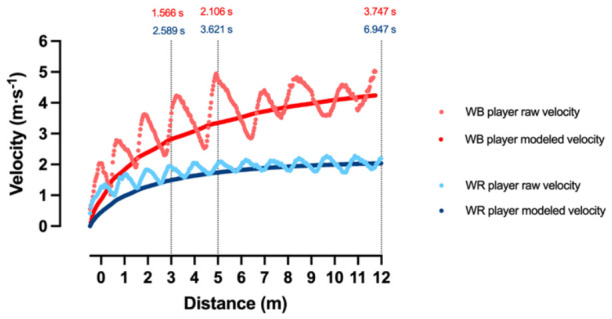
Raw (pale red and blue) and modelled (solid dark red and blue) wheeling velocity profiles (m·s^−1^) for one wheelchair basketball player and one wheelchair rugby player. Velocity data were collected with a linear position transducer and fitted with a mono-exponential model using least squares regression (Equation (1)). Vertical dashed lines denote the 3, 5, and 12 m split times derived from the modelled displacement–time curves.

In the 12 m linear wheeling sprint test, between-group differences became progressively larger as sprint distance increased. Large, significant differences were observed at both 5 m (mean ± SD = 2.104 ± 0.227 s vs. 2.647 ± 0.449 s; *p* < 0.001; ES = 1.53) and 12 m (3.829 ± 0.335 s vs. 4.963 ± 0.892 s; *p* < 0.001; ES = 1.68), with athletes representing higher functional performance profiles achieving faster sprint times. The magnitude of these differences suggests that short-distance sprint performance, particularly beyond the initial acceleration phase, is sensitive to functional differences affecting propulsion efficiency and trunk control (Figure 3).

Finally, in the wheelchair manoeuvrability test (3L3R), moderate but significant differences were observed in total completion time (sum of left- and right-turn splits) between groups (mean ± SD = 19.446 ± 1.392 s vs. 21.396 ± 4.21 s; *p* = 0.043; ES = 0.62). However, no significant differences were found for the right-turn split time (9.684 ± 0.744 s vs. 10.448 ± 2.307 s; *p* = 0.095; ES = 0.45). These findings indicate that although overall manoeuvrability performance differed moderately between groups, isolated turning performance showed smaller differences, suggesting that the 3L3R test may have lower discriminative sensitivity compared with propulsion and sprint assessments.

## 4. Discussion

The present study examined the discriminative capacity of commonly used field-based wheelchair performance tests in elite wheelchair court athletes presenting different functional performance profiles. The main findings indicate that propulsion (IMPRP) and sprint tests demonstrate strong sensitivity to differences in propulsion capacity between athlete groups, whereas manoeuvrability performance (3L3R) shows only moderate discriminative ability. These results suggest that propulsion-based assessments may represent particularly informative tools for monitoring propulsion-related performances in wheelchair court sports.

The IMPRP test produced the most pronounced differences between groups, with athletes representing higher functional performance profiles achieving greater propulsion velocities, power, and force outputs. These findings are consistent with previous research linking propulsion performance with functional classification and upper-limb capability in wheelchair athletes [20]. Athletes with greater trunk control and upper-limb function are able to generate more effective push-rim force and maintain more efficient propulsion mechanics, which likely explains the substantially higher mechanical outputs observed. Because the IMPRP requires a single maximal bilateral propulsion effort with minimal corrective movement, it appears particularly sensitive to limitations in trunk stability, hand function, and force transmission [12,20]. The magnitude of these differences (ES ≥ 1.75) indicates very large practical effects, suggesting that propulsion capacity differs substantially between the functional performance profiles represented in this study.

Sprint performance also clearly differentiated the athlete groups. The progressively larger between-group differences observed from 5 m to 12 m may indicate that propulsion-related performance characteristics become increasingly influential across repeated propulsion cycles. These findings are consistent with previous literature showing strong relationships between sprint performance and wheelchair sport performance characteristics in elite athletes [5]. Short-distance wheelchair sprint tests, therefore, appear particularly useful for capturing dynamic propulsion capacity, which integrates both mechanical force production and wheelchair handling efficiency during repeated push cycles.

In contrast, manoeuvrability performance assessed through the 3L3R test showed smaller between-group differences. Although total completion time differed moderately between groups, individual turning phases did not consistently show significant differences. This finding may indicate that manoeuvrability performance depends not only on propulsion capacity but also on technical skill, wheelchair configuration, and sport-specific tactical adaptations. Elite athletes often develop highly refined wheelchair handling strategies that may partially compensate for functional limitations, potentially reducing the sensitivity of manoeuvrability tests to underlying performance-related differences. Consequently, manoeuvrability assessments may be more informative for evaluating technical proficiency rather than core propulsion capacity.

Importantly, the present results should not be interpreted simply as differences between sports. Rather, they demonstrate that commonly used field-based propulsion and sprint tests are sensitive to functional performance differences associated with distinct impairment profiles within wheelchair court athletes. This observation is particularly relevant given the ongoing need for objective, quantifiable performance assessments capable of complementing existing observational approaches in Paralympic sport evaluation systems [25,26].

The reliability analyses further support the suitability of these assessments for applied use. Across all tests, reproducibility was high, with ICC values generally exceeding 0.82 and low CVs. Such reliability is essential when implementing field-based assessments within high-performance environments, where practitioners require stable and sensitive tools to monitor changes in athlete performance across training cycles.

Several limitations should be acknowledged. The relatively small sample size reflects the limited availability of elite Paralympic athletes but may restrict the generalizability of the findings. However, considering the present sample size, the large effect sizes observed in most IMPRP and sprint variables suggest acceptable statistical sensitivity for detecting meaningful between-group differences. In contrast, the lower ES and statistical power observed in the 3L3R test indicate that these findings should be interpreted with greater caution. Additionally, the use of sport-specific wheelchairs may have influenced propulsion and manoeuvrability performance, as wheelchair configuration differs between sports, stability, camber, and contact-oriented design. While this approach enhances ecological validity, it also prevents isolating the specific contribution of equipment-related factors to propulsion performance. Future research should aim to include larger multi-national cohorts and combine field testing with biomechanical analyses to further clarify the relative contribution of functional characteristics, sport-specific demands, wheelchair configuration, and propulsion mechanics to wheelchair court sport performance.

Despite these limitations, the present study provides novel insight into the discriminative capacity of widely used field-based wheelchair performance tests. The findings indicate that propulsion and sprint assessments are particularly sensitive to propulsion-related performance differences between the sampled athlete groups, whereas manoeuvrability tests may be less suitable for this purpose. These observations support the continued use of propulsion and sprint testing within athlete monitoring frameworks and highlight their potential value for informing objective evaluation approaches in Paralympic sport.

## 5. Practical Applications

From an applied perspective, incorporating IMPRP and sprint testing into athlete monitoring routines appears valuable in wheelchair court sports. These assessments provide reliable and time-efficient indicators of propulsion capacity that can assist coaches and performance staff in tracking physical development and guiding individualised training interventions.

In addition, the sensitivity of propulsion and sprint tests to between-group performance differences suggests that they may contribute objective information for athlete profiling and performance monitoring in wheelchair court sport contexts. In contrast, manoeuvrability tests such as the 3L3R may be more appropriate for assessing sport-specific wheelchair handling and technical proficiency rather than underlying propulsion capacity.

## 6. Conclusions

The present study showed that field-based propulsion and sprint tests present acceptable-to-high reliability and are sensitive to between-group performance differences in elite wheelchair court athletes. IMPRP and short-distance sprint tests demonstrated the strongest discriminatory patterns, whereas manoeuvrability performance showed more moderate between-group differences. However, these findings should be interpreted within the context of ecologically valid wheelchair court sport groups, as sport-specific demands, wheelchair configuration, propulsion strategies, and training background may also contribute to performance outcomes. Collectively, these assessments appear useful for athlete profiling and performance monitoring in wheelchair court sports, although future studies using more controlled designs are required before extending these findings toward broader functional evaluation frameworks.

## Figures and Tables

**Table 1:** Physical characteristics and functional categories of elite wheelchair court athletes.

Player	Age (Years)	Sport	Total Mass (kg) (BM + WM)	IWBF Class	WWR Class	A-B-C-D Category
1	26	Wheelchair Basketball	83	3.5	--	B
2	26	Wheelchair Basketball	64.4	3.0	--	B
3	49	Wheelchair Basketball	99.3	3.0	--	B
4	21	Wheelchair Basketball	104.4	4.5	--	B
5	24	Wheelchair Basketball	82.9	4.0	--	B
6	20	Wheelchair Basketball	51.4	1.5	--	A
7	23	Wheelchair Basketball	83.6	3.5	--	B
8	42	Wheelchair Basketball	68.4	2.5	--	A
9	20	Wheelchair Basketball	101.2	3.0	--	B
10	27	Wheelchair Basketball	91	4.5	--	B
Total	27.8 ± 9.8		83.0 ± 17.3			
11	41	Wheelchair Rugby	80	--	0.5	D
12	30	Wheelchair Rugby	88.5	--	2.5	C
13	32	Wheelchair Rugby	104.7	--	1.5	D
14	49	Wheelchair Rugby	85.3	--	3.0	C
15	36	Wheelchair Rugby	77	--	3.0	C
16	31	Wheelchair Rugby	55	--	2.5	C
17	29	Wheelchair Rugby	82	--	2.0	C
18	25	Wheelchair Rugby	81.1	--	1.0	D
19	45	Wheelchair Rugby	98	--	0.5	D
Total	35.3 ± 8.1		83.5 ± 14.0			

BM = body mass; WM = wheelchair mass; IWBF = International Wheelchair Basketball Federation; WWR = World Wheelchair Rugby; wheelchair basketball players were grouped into lower (A: 1.0–2.5) and higher (B: 3.0–4.5) functional categories. Wheelchair rugby players were grouped into lower (D: 0.5–1.5) and higher (C: 2.0–3.5) functional categories.

**Table 2:** Intraclass reliability values of wheelchair basketball players in initial maximum push-rim propulsion (IMPRP), wheelchair manoeuvrability test (3L3R), and sprint tests.

	Trial 1	Trial 2	Change in Mean	Lower and Upper Confident Limits	ICC	Lower and Upper Confident Limits	CV (%)	Typical Error
**IMPRP**								
V (m·s^−1^)	1.11 ± 0.20	1.08 ± 0.19	−0.03	−0.07–0.01	0.95	0.84–0.98	3.64 ± 3.93	0.05
Vmax (m·s^−1^)	2.38 ± 0.27	2.34 ± 0.29	−0.04	−0.09–0.01	0.96	0.88–0.99	1.88 ± 2.72	0.06
P (W)	262.55 ± 107.11	248.94 ± 84.09	−13.61	−32.24–5.02	0.96	0.88–0.99	5.51 ± 5.74	22.72
Rel. P (W·Kg^−1^)	3.14 ± 0.90	3.03 ± 0.80	−0.11	−0.31–0.08	0.94	0.82–0.98	5.51 ± 5.74	0.24
Pmax (W)	812.32 ± 259.27	772.04 ± 215.05	−40.28	−122.35–41.80	0.86	0.62–0.95	9.30 ± 6.93	100.12
Rel. Pmax (W·Kg^−1^)	9.78 ± 2.27	9.36 ± 2.19	−0.41	−1.28–0.46	0.82	0.52–0.94	9.30 ± 6.93	1.06
F (N)	212.69 ± 64.95	206.30 ± 52.69	−6.40	−15.90–3.10	0.97	0.91–0.99	3.57 ± 3.55	11.59
Rel. F (N·Kg^−1^)	2.57 ± 0.55	2.53 ± 0.54	−0.04	−0.15–0.07	0.96	0.86–0.99	3.57 ± 3.55	0.13
Fmax (N)	432.78 ± 81.79	431.20 ± 82.73	−1.58	−21.84–18.69	0.93	0.80–0.98	4.44 ± 3.17	24.72
Rel. Fmax (N·Kg^−1^)	5.29 ± 0.73	5.24 ± 0.57	−0.05	−0.28–0.19	0.85	0.58–0.95	4.44 ± 3.17	0.29
**Sprint**								
3 m (s)	1.60 ± 0.15	1.60 ± 0.23	0.00	−0.07–0.07	0.85	0.60–0.95	3.75 ± 3.15	0.08
5 m (s)	2.16 ± 0.18	2.16 ± 0.24	0.00	−0.07–0.06	0.91	0.74–0.97	2.49 ± 2.01	0.07
12 m (s)	3.94 ± 0.31	3.91 ± 0.36	−0.02	−0.09–0.04	0.95	0.86–0.99	1.64 ± 1.31	0.08
**3L3R**								
L	10.13 ± 0.90	9.98 ± 0.65	−0.14	−0.33–0.04	0.94	0.81–0.98	1.71 ± 1.49	0.23
R	10.0 ± 0.84	9.80 ± 0.72	−0.20	−0.42–0.03	0.91	0.73–0.97	2.20 ± 2.06	0.27
Total	20.13 ± 1.67	19.79 ± 1.36	−0.34	−0.68–0.00	0.94	0.83–0.98	1.78 ± 1.39	0.42

**Table 3:** Intraclass reliability values of wheelchair rugby players in initial maximum push-rim propulsion (IMPRP), wheelchair manoeuvrability test (3L3R), and sprint tests.

	Trial 1	Trial 2	Change in Mean	Lower and Upper Confident Limits	ICC	Lower and Upper Confident Limits	CV (%)	Typical Error
**IMPRP**								
V (m·s^−1^)	0.74 ± 0.20	0.73 ± 0.13	−0.01	−0.08–0.06	0.82	0.50–0.95	6.91 ± 6.09	0.08
Vmax (m·s^−1^)	1.40 ± 0.34	1.41 ± 0.34	0.02	−0.02–0.05	0.99	0.98–1.00	2.29 ± 1.60	0.04
P (W)	97.09 ± 34.16	100.54 ± 44.28	3.45	−4.18–11.07	0.97	0.89–0.99	8.64 ± 7.54	8.70
Rel. P (W·Kg^−1^)	1.26 ± 0.60	1.30 ± 0.73	0.04	−0.07–0.15	0.98	0.92–0.99	8.64 ± 7.54	0.12
Pmax (W)	231.72 ± 110.29	262.13 ± 159.52	30.41	−5.84–66.66	0.94	0.79–0.98	10.93 ± 6.92	41.36
Rel. Pmax (W·Kg^−1^)	3.00 ± 1.68	3.38 ± 2.18	0.39	−0.04–0.81	0.96	0.86–0.99	10.93 ± 6.92	0.49
F (N)	124.91 ± 35.59	132.05 ± 43.50	7.40	−1.82–16.11	0.95	0.85–0.99	7.67 ± 6.83	10.23
Rel. F (N·Kg^−1^)	1.58 ± 0.46	1.68 ± 0.60	0.10	−0.03–0.22	0.95	0.83–0.98	7.67 ± 6.83	0.15
Fmax (N)	241.25 ± 86.22	265.99 ± 102.53	24.74	4.32–45.16	0.96	0.86–0.99	7.98 ± 7.82	23.30
Rel. Fmax (N·Kg^−1^)	3.04 ± 1.02	3.38 ± 1.31	0.34	0.04–0.63	0.94	0.81–0.98	7.98 ± 7.82	0.34
**Sprint**								
3 m (s)	1.96 ± 0.32	1.97 ± 0.29	0.01	−0.04–0.07	0.97	0.90–0.99	2.58 ± 2.12	0.07
5 m (s)	2.77 ± 0.46	2.79 ± 0.41	0.02	−0.05–0.09	0.98	0.92–0.99	2.27 ± 1.77	0.08
12 m (s)	5.25 ± 0.95	5.27 ± 0.85	0.02	−0.08–0.12	0.99	0.96–1.00	1.65 ± 1.20	0.11
**3L3R**								
L	11.79 ± 1.92	11.74 ± 1.91	−0.05	−0.11–0.00	1.00	1.00–1.00	0.51 ± 0.40	0.07
R	11.68 ± 2.47	11.54 ± 2.36	−0.14	−0.35–0.07	0.99	0.98–1.00	1.61 ± 1.05	0.24
Total	23.47 ± 4.39	23.26 ± 4.22	−0.20	−0.41–0.01	1.00	0.99–1.00	0.76 ± 0.72	0.24

V = mean velocity; Vmax = maximum velocity; P = mean power; Rel. P = relative mean power; Pmax = maximum power; Rel. Pmax = relative maximum power; F = mean force; Rel. F = relative mean force; Fmax = maximum force; Rel. Fmax = relative maximum force; ICC = intraclass correlation coefficient; CV = coefficient of variation. No significant differences were found between Trial 1 and Trial 2.

## Data Availability

The data are not publicly available due to privacy and ethical restrictions.
